# Structure Prediction and Mechanical Properties of Silicon Hexaboride on Ab Initio Level

**DOI:** 10.3390/ma14247887

**Published:** 2021-12-20

**Authors:** Tamara Škundrić, Branko Matović, Aleksandra Zarubica, Jelena Zagorac, Peter Tatarko, Dejan Zagorac

**Affiliations:** 1 Materials Science Laboratory, Vinča Institute of Nuclear Sciences, University of Belgrade, 11351 Belgrade, Serbia; tamara.skundric@vinca.rs (T.Š.); mato@vinca.rs (B.M.); jelena@vinca.rs (J.Z.); 2 Center for Synthesis, Processing and Characterization of Materials for Application in the Extreme Conditions “Cextreme Lab”, University of Belgrade, 11001 Belgrade, Serbia; 3 Department of Chemistry, Faculty of Sciences and Mathematics, University of Nis, 18000 Nis, Serbia; zarubica2000@yahoo.com; 4 Institute of Inorganic Chemistry, Slovak Academy of Sciences, 845 36 Bratislava, Slovakia; peter.tatarko@savba.sk

**Keywords:** SiB_6_, silicon hexaboride, data mining, elastic properties, DFT

## Abstract

Silicon borides represent very appealing industrial materials for research owing to their remarkable features, and, together with other boride and carbide-based materials, have very wide applications. Various Si–B phases have been investigated in the past, however a limited number of studies have been done on the pristine SiB_6_ compound. Structure prediction using a data mining ab initio approach has been performed in pure silicon hexaboride. Several novel structures, for which there are no previous experimental or theoretical data, have been discovered. Each of the structure candidates were locally optimized on the DFT level, employing the LDA-PZ and the GGA-PBE functional. Mechanical and elastic properties for each of the predicted and experimentally observed modifications have been investigated in great detail. In particular, the ductility/brittleness relationship, the character of the bonding, Young’s modulus E, bulk modulus B, and shear modulus K, including anisotropy, have been calculated and analyzed.

## 1. Introduction

Silicon borides are lightweight ceramic compounds formed between silicon and boron and are regarded as the most elusive refractory compounds [1]. Metal borides, especially silicon borides, are very appealing industrial materials with potentially very wide applications. Due to silicon boride’s extraordinary features, it represents a very promising material for future research, and has excellent electrical conductivity [2], a high degree of hardness [2,3], a moderate melting point (2123 K), and a low specific gravity [2,3]. According to the literature, there are a few previously reported phases in the silicon boron system: SiB_3_, SiB_6_, and SiB_n_ [4,5,6]. Silicon hexaboride was first reported in 1900 by Henry Moissan and Alfred Stock after briefly heating silicon and boron in a clay vessel, and was described as black, irregular crystals [7]. As it is known, one of the first reports of this structure described it as a cubic structure *Pm-3m* (no. 221) [8], but later confirmed it to be mechanically unstable [9]. Later studies presented SiB_6_ as an orthorhombic structure [1,10], combined with interconnected icosahedra, icosihexahedra, and several isolated boron and silicon atoms [10]. Based on first-principle calculations and CALYPSO structure prediction methodology, two structure candidates, defined as mechanically stable, have been proposed: monoclinic *P21/m* and hexagonal *R3-m* structure types [9]. Furthermore, the first of these newly proposed structures, *P21/m*-SiB_6_, is found to be an indirect band gap of 0.41 eV, while *R3m*–SiB_6_-81, has a direct band gap of 1.654 eV. There is also an indication that this new theoretically suggested material might be useful as a refractory n-type semiconductor capable of operating in extreme environments [9].

First-principles molecular dynamics (MD) simulations revealed pentagonal pyramid-like motifs and B12 icosahedral molecules as the key structural unit of the amorphous network. This amorphous silicon hexaboride exhibited semiconducting behavior with a theoretical bandgap energy of 0.3 eV [11]. It was suggested that irradiation leads to the structural change from crystalline to an amorphous state. A low irradiation dose retained the crystalline structure, with an increase in amorphization being observed with an increase in the irradiation dose [12]. Unirradiated SiB_6_ under atmospheric pressure and room temperature have an orthorhombic symmetry and crystalline structure of the *Pnnm* spatial group, but, subsequent to the irradiation, a change in the lattice parameters was observed [13]. An increase in irradiation dose had an inverse effect on the activation energy and a directly proportional effect on the lattice volume, although the enlargement of the cell is considered to be due to stress that the samples experienced, caused by an increase in the gamma irradiation dose [13]. Gamma irradiation also leads to a large number of defects, which then leads to increased oxidation in the material, which, in unirradiated samples, takes place at relatively low temperatures, compared to the irradiated samples, where oxidation occurs at higher temperatures [14].

There is also a report of a pressure-induced phase transformation of amorphous silicon hexaboride SiB_6_, where α-SiB_6_ undergo a gradual phase transformation to a high-density amorphous phase (HAD) which consists of differently coordinated motifs ranging from four to eight, but B12 icosahedra are found to be persist during the compression of this phase [15]. Upon pressure release, an amorphous structure could be recovered [15]. With the assumption of the fixed configuration of boron and silicon, according to some earlier research, atoms in B_6_Si have a definite density of states at its Fermi level [16]. Dynamically and mechanically stable at ambient pressure, a novel metallic silicon hexaboride, *Cmca*-SiB_6_, is proposed, with lower formation energy compared to the synthesized *Pnnm*-SiB_6_, which suggests its thermodynamic stability. Electronic structure analysis revealed the novel two-dimensional metallicity of this phase [17]. Moreover, negative formation Gibbs energy of the SiB_6_ confirms the thermodynamic stability of the phase [18].

Regardless of the excellent properties of SiB_6_, its application has been limited as a result of the poor sinterability using conventional sintering techniques. However, new sintering methods have been proposed. It was found that a rare earth element (La) was very effective in evolving the microstructure of the SiB_6_ phase, resulting in reducing the sintering temperature, controlling grain growth, and improving the crystallinity of the SiB_6_ grain [19]. There is also a report of prepared Si–B films from mixed gasses of silane and diborane as source materials by chemical vapor deposition (CVD) using high-frequency induction heating, whereby is suggested that, at a lower B/Si ratio in the source gas, SiB_4_ was formed, while SiB_6_ could be also formed at the higher ratios of B/Si [20]. According to some earlier research, increasing the sintering temperature lead to an increase in the relative density of SiB_6_, and was above 99% at a 1923 K sintering temperature, and X-ray analysis confirmed that SiB_6_ was the only material present [2]. Increased temperature also affects electrical conductivity and the Seebeck coefficient, which increase, apart from thermal conductivity, which decreases [2]. There is also a suggestion that the “chemical oven” method can be used as a simple and effective method to obtain infrared ceramic SiB_6_ [21].

Recent studies have revealed that the nano-SiB_6_ particles that were physically mixed into pentaerythritol PE as nucleating agents lead to the formation of a composite that has larger specific heat, lower solid–solid phase change temperature, and latent heat than that of pure PE [22]. According to the latest research, silicon hexaboride can enhance the self-healing performance of MoSi_2_ ceramic, as the introduction of the SiB_6_ phase improved the oxidation resistance of MoSi_2_ coatings at low–medium temperatures [23]. There is also an indication that electrical and thermal conductivity and Seebeck coefficient in the Si–B system largely depends on the processing routes, along with the boron content of the system [18]. Due to its features, the silicon–boron system has been quite well-researched to examine its potential as a high-temperature material. Hence, it was suggested that SiB_6_ exhibits very good thermoelectric material characteristics at high temperatures [2,24]. There have also been novel proposed synthesis methods for these surface-protected, oxidation-resistant semiconductor materials within the Si–B system, and these materials are considered to be very useful for various high temperature solar thermal, or solar electric applications [25]. Nowadays, studies are also focused on investigating the properties of SiB_6_ compounds for their use in nanotechnology applications [25].

## 2. Computational Methods

Our general approach to performing structure prediction and gaining insight into the structural stability of possible phases existing in the SiB_6_ system is based on a data mining search. We performed data-mining-based explorations of the ICSD database [26,27] via resemblance to known crystallographic structures. According to some earlier studies, a data mining search could be also used as an additional method for some others, such as global optimization, for instance, because it has proven successful in finding additional possible modifications in some chemical systems. Additionally, many relevant structure candidates in a given chemical system exhibited a very similar crystal structure to some other compounds observed in another chemical system, even though there was no obvious chemical relationship between these two chemical systems [28]. We have used the well-known knowledge discovery in databases (KDD) process, which involves selection, preprocessing, transformation, and interpretation/evaluation (or post-processing), and it has been already used successfully in some previous studies [29,30,31]. All potential structure candidates that appear in the ICSD database have been extracted and subsequently submitted to local optimization at an ab initio level. Details about the KDD process and the combination of data mining with ab initio methods can be found elsewhere [32,33]. After the structure, candidates were identified using the data mining approach, and are submitted to density functional theory (DFT) calculations. Local optimizations (including the cell parameters and atom positions) were performed using the CRYSTAL17 code [34,35,36], which is based on linear combinations of atomic orbitals (LCAO). Structure optimizations were performed on the DFT level, employing two different functionals: the Generalized Gradient Approximation (GGA) with the Perdew–Burke–Ernzerhof (PBE) functional [37] and the Local Density Approximation (LDA) with the Perdew–Zunger (PZ) correlation functional [38] for comparison. An all-electron basis set based on Gaussian-type orbitals was employed; in particular, in the case of Silicon a *[5s4p1d]* basis set was used [39,40], and the *[3s2p1d]* basis set was used in the case of boron [41,42,43] (further basis sets information are given in the Appendix B). In each structural optimization, Fock/KS matrix mixing was set to 30%, and the tolerances for the convergence on energy were set to 10^−7^. K-point meshes of 8 × 8 × 8 Monkhorst-Pack scheme have been used. Chosen LCAO/Gaussian basis set type approach and DFT methods have been shown highly efficient and precise in our previous theoretical studies [44,45,46] and in comparison to the experimental data [30,47,48]. A computational strategy implemented in the CRYSTAL17 solid-state, quantum-chemical program has been performed for the accurate ab initio simulation of elastic and mechanical properties of crystalline materials [49]. Full elastic tensor has been generated using the keyword ELASTCON [50]. Elastic tensor analysis and visualization have been performed using ELATE code [51]. The symmetries of the analyzed structures were determined using the SFND [52] and RGS [53] algorithms implemented in the program KPLOT [54]. The structures were visualized using the Vesta3 program [55].

## 3. Results and Discussion

### 3.1. Structure Prediction of Silicon Hexaboride

The data mining searches were performed within the ICSD database, which included more than 250,000 crystal structures in the latest release, with more than 180,000 structures assigned to 9873 distinct structure types [26,27]. In order to find new structures in the SiB_6_, data mining-based searches to find all possible A_6_X structure types in the ICSD database [20,21] have been used. In particular, the data mining has resulted in the previously investigated silicon hexaboride structures: the c-SiB_6_ (or CaB_6_) type, the SiB_6_(*Cmca*) type, the SiB_6_(*P21/m*) type, the SiB_6_-81 (*R3m*) type, the SiB_6_(*Pnnm*) type, and the following AX_6_ structure types, which together could be used in search of another chemical system: the PB_6_ (or α-B_6_O) type, the β-B_6_O type the BaN_6_ type, the BaSi_6_ type, the HgN_6_ type, the OsOF_5_ type, the Al_6_Mn type, the Ga_6_Pu type, the MoCl_6_ (*P-3m1)* type, the MoCl_6_ (*P-3c1)* type, the Au_6_Sm type, the MnU_6_ type, the PbN_6_ (*Pna21*) type, the PbN_6_ (*Pcmn*) type, the RbTe_6_ type, the *c*-SF_6_ type, the *LT*-SF_6_ type, the SF_6_ (*C-1*) type, the MoZn_6_ type, the SrN_6_ type, the TeOH_6_ type, the CeCu_6_ type, the WCl_6_ (*R-3H*) type, the WCl_6_ (*P-3m1*) type, the XeF_6_ (*P121/c1*) type, the XeF_6_ (*C12/c1*) type, the XeF_6_ (*Pc21n*) type, the PrAu_6_ type, the Cu_6_Nd type, and the UCl_6_ type.

Nine additional structure candidates have been created using the Primitive Cell approach for Atom Exchange (PCAE) method [31,56], despite resulting in non-stoichiometric compounds. Since the above-mentioned prototypes are not common, data mining and PCAE based searches resulted in 44 structure candidates. A total number of structure candidates were finally reduced after performing full structural optimization at the ab initio level, and four final structures were distinguished as being the most energetically favorable options, regardless of the computational approach (Table 1). It is not surprising to find a large quantity of the energetically non-favorable structures, since many of the prototypes from the data mining-based search resulted in several non-equilibrium structures in the SiB_6_ and other parent compounds, or in non-stoichiometric compounds, as previously observed [9,10,57,58].

The most relevant structure candidates predicted for the SiB_6_ compound are α-SiB_6,_ β-SiB_6_, γ-SiB_6_, and δ-SiB_6_ modifications (Table 1). Full structural data of all favorable candidates are given in Table 2 for calculations with the PBE functional, and those computed with the LDA functional are shown in Table A1. Table 3 presents structure details and unit cell parameters of the four predicted candidates chosen for further theoretical analysis in comparison to the previous experimental and theoretical results where available. Besides the experimentally known cubic SiB_6_, the orthorhombic phase in space group *Cmce* (no. 64) has been recently predicted [17], and the results obtained within this study are in good agreement with those for both known structures on the GGA-PBE and LDA-PZ levels of calculation. We noted that, in both previous theoretical calculations involving SiB6 structures DFT (GGA-PBE) and CASTEP, code has been used based on the robust methods of a plane-wave (PW) basis set and pseudopotentials (PPs) (Table 3 [9,17]). Our LCAO-GGA-PBE calculations concur with these PW/PPs-GGA-PBE data, as we expect to agree with possible PAW/PPs-GGA-PBE and FP/APW + LO-PBE calculations [59]. Moreover, we have predicted two additional structures denoted as α- and δ- SiB_6_ modifications, for which there are no previous data, but the results of structural relaxation agree between two levels of calculations (GGA-PBE and LDA-PZ).

**Figure 1:**
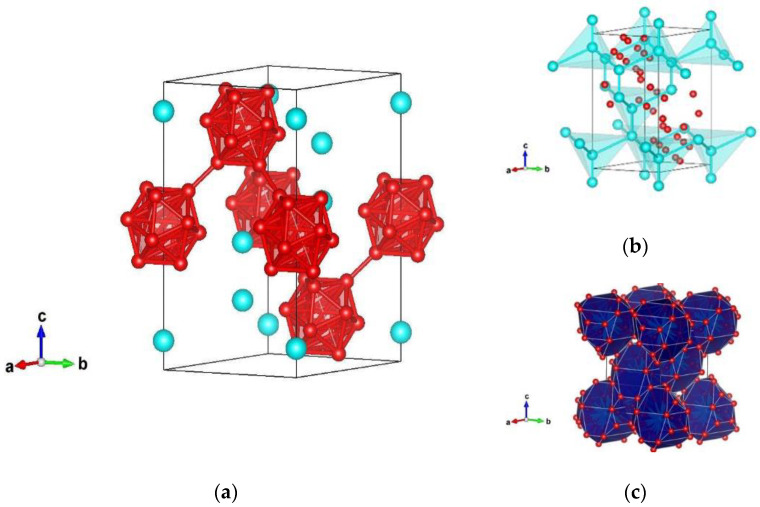
Visualization of the α-SiB_6_-type modifications in the space group *R-3mH* (no. 166) represented with: (**a**) B–B atoms; (**b**) Si–Si atoms; (**c**) Si–B atoms in the second coordination polyhedra. Blue and red spheres denote Si and B atoms, respectively.

The data mining ab initio method resulted in four final structure candidates in the SiB_6_ system. The energetically most favorable modification after local optimization is denoted as α-SiB_6_-type and appears in hexagonal space group *R-3mH* (no. 166) with unit cell parameters of a = 6.164 Å and c = 12.079 Å on the GGA-PBE level of calculation. The α-SiB_6_ phase is visualized in Figure 1, while full structural data are presented in Table 2 for calculation with the PBE functional, and in Table A1 when computed with the LDA functional, respectively.

From the literature data, it appears that B12 icosahedra, as the single primary unit of α-rhombohedral boron, is a fundamental structural element for most of the Si–B compounds [9,60]. This is the case in the α-SiB_6_ phase, with the PB_6_ structure type, as in the case of the α-B_6_O compound [46,58,61,62]. Boron atoms form B12 icosahedra with interatomic distances from 1.73 Å to 2.95 Å, as shown in Figure 1a. Moreover, silicon atoms in the second coordination polyhedra (CP) form corner-connected tetrahedra with Si–Si distances 1 × 2.448 Å and 3 × 3.893 Å (Figure 1b). On the other hand, when analyzing Si–B distances, the silicon atom is surrounded by only three boron atoms in the first CP (3 × 2.005 Å), and with 30 boron atoms in the second CP, creating a complex polyhedra, shown in Figure 1c.

**Figure 2:**
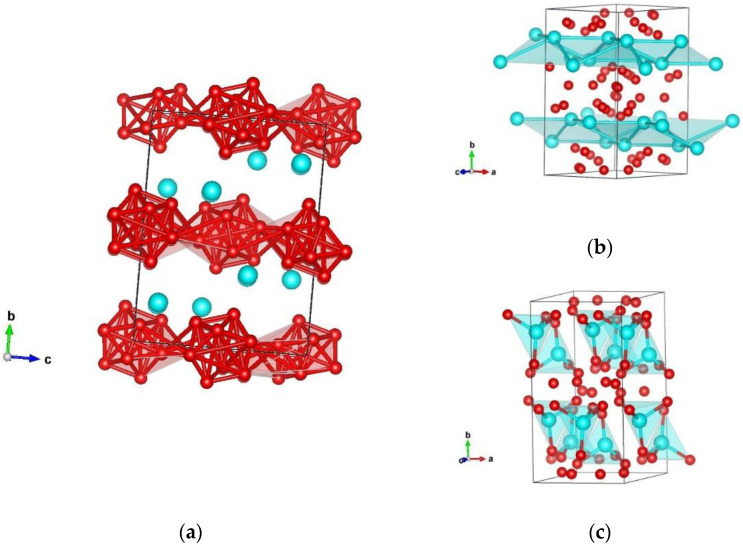
Visualization of the orthorhombic β-SiB_6_-type structure in space group *Cmce* (no. 64) represented with (**a**) B–B atoms; (**b**) Si–Si atoms; (**c**) Si–B atoms in the second coordination polyhedra. Blue and red spheres denote Si and B atoms, respectively.

A second, energetically favorable structure candidate, found through the data mining approach, is referred to as a β-SiB_6_-type of modification. It crystallizes in space group *Cmce* (no. 64) with unit cell parameters a = 5.894, b = 11.184, and c = 8.420 Å (GGA), and it has five non-equivalent Si1, B1, B2, B3, and B4 atoms in the structure, for which full structural data are given in Table 2. This orthorhombic structure is consisted of three layers of boron icosahedra within the unit cell, with layers of silicon atoms between them. As in the previous α-SiB_6_-phase, boron atoms within this β-modification form four distinct B12 icosahedra with atom–atom distances from 1.73 Å to 2.87 Å, and the structure is visualized in Figure 2a. Besides, in the second coordination polyhedra (CP), silicon atoms are in four-fold coordination with interatomic distances of 2 × 3.638 Å and 2 × 3.977 Å, respectively (Figure 2b). Upon examination of Si–B bonding, it appears that, within the first coordination polyhedra (CP), the silicon atom is surrounded by four boron atoms, thus forming a tetrahedra with interatomic distances of 1 × 2.021 Å–B, 1 × 2.064 Å–B, and 2 × 2.085 Å–B, which is visualized in Figure 2c.

**Figure 3:**
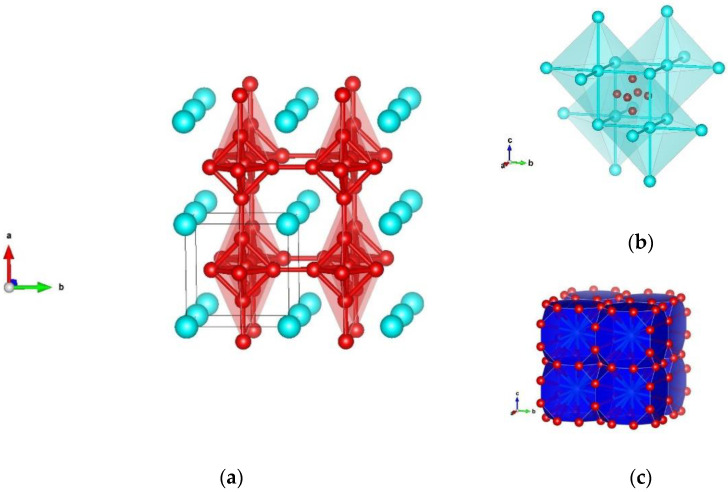
Visualization of γ-SiB_6_-type of modification in space group *Pm-3m* (no. 221) represented with: (**a**) B–B atoms; (**b**) Si–Si atoms; (**c**) Si–B atoms in the second coordination polyhedra. Blue and red spheres denote Si and B atoms, respectively.

Maybe the most investigated structure in the silicon hexaboride, both experimentally and theoretically, is found as a cubic phase, denoted as γ-SiB_6_-type. It appears in space group *Pm-3m* (no. 221), while full structural data are given in Table 1, Table 2 and Table A1. Previous reports show unit cell parameters of a = 4.130 Å, which concur very well with our GGA (a = 4.161 Å) and LDA (a = 4.109 Å) results (Table 1). First reports of the SiB_6_ compound described it as a cubic phase and indicated that it is isomorphous with CaB_6_ [8]. Hence, within this γ-modification, boron atoms have five-fold coordination, with interatomic distances of 1 × 1.663 Å and 4 × 1.766 Å, and the structure is visualized in Figure 3a. Additionally, in the second coordination polyhedra (CP), silicon atoms form octahedra with atom–atom distances of 6 × 4.161 Å, which is visualized in Figure 3b. Furthermore, analyzing Si–B distances of silicon atoms were found to be surrounded by 24 boron atoms, with the distance between the atoms being 24 × 3.057 Å–B, as shown in Figure 3c. In addition, it has been confirmed that there is ionic bonding in the isomorphous CaB_6_ phase between the boron group and the Ca atom [63].

**Figure 4:**
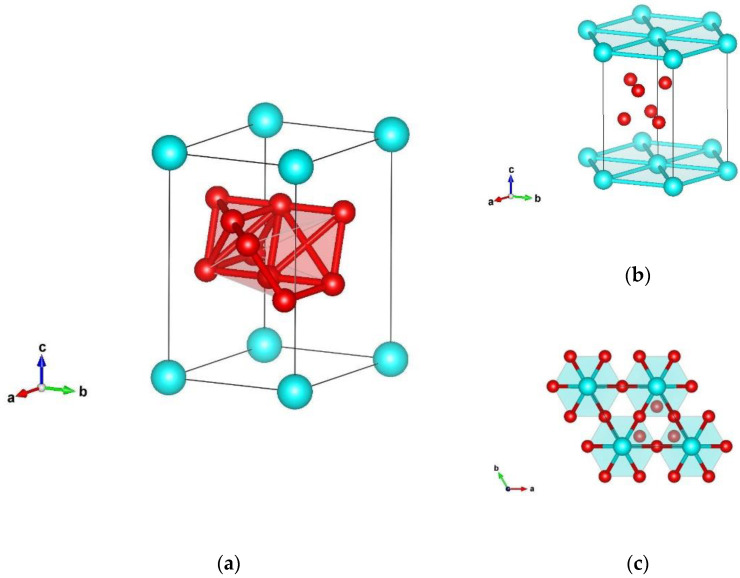
Visualization of δ-SiB_6_-type (hexagonal axes) in space group *P3m1* (no. 156) represented with (**a**) B–B atoms; (**b**) Si–Si atoms; (**c**) Si–B atoms. Blue and red spheres denote Si and B atoms, respectively.

The last modification of four yielded from our searches was the rhombohedral structure, denoted as δ-SiB_6_-type, that crystallizes in space group *P3m1* (no. 156) with unit cell parameters a = 3.503 and c = 6.407 Å (GGA). Besides silicon, there are two different boron atoms, B1 and B2, and full structural data are listed in Table 2 (LDA in Table A1). Within δ-SiB_6_-type of the structure represented with hexagonal axes, boron atoms are in six-fold coordination and form two distinct octahedra with atom–atom distances (B1 2 × 1.736 Å–B, 2 × 1.766 Å–B, 2 × 1.952 Å–B, B2 2 × 1.749 Å–B, 2 × 1.754 Å–B, 2 × 1.952 Å–B). Within δ-modification, six-fold coordinated silicon atoms with interatomic distances of 6 × 3.503 Å form a layered-like structure, with boron octahedra situated between these two layers (Figure 4b). In addition, when examining Si–B distances, silicon atoms were surrounded by six boron atoms, with a distance between atoms of 6 × 2.479 Å–B, which is visualized in Figure 4c.

### 3.2. Elastic and Mechanical Properties of SiB_6_

Surprisingly, there is a limited number of investigations on the elastic and mechanical properties of SiB_6_, both in theory and in experiment. Experimental work is mostly devoted to non-stochiometric SiB_6_ or doped compounds [3,64,65,66,67], while there are only a few recent theoretical studies on silicon hexaboride [9,11,17]. In this study, the elastic constants *Cij* for the most relevant silicon hexaboride modifications (α-SiB_6_, β-SiB_6_, and γ-SiB_6_) have been calculated using a GGA-PBE and LDA-PZ approach and were compared to previous theoretical data where found. Cubic γ-SiB_6_ modification has only three independent elastic constants, namely *C*_11_, *C*_12_, and *C*_44_, and they are given in Table 4 and Table A2. These calculated elastic constants are in good agreement with available theoretical data from Ref. [9] (Table 4). 

Using the elastic constants, one can calculate the mechanical stability of the corresponding modifications using the mechanical stability criteria [68,69]. There are three conditions for cubic crystals:

*C*_44_ > 0; *C*_11_ − *C*_12_ > 0; *C*_11_ + 2 *C*_12_ > 0,

The results of the elastic constants (Table 4 and Table A2) are not satisfying one of the mechanical stability criteria, since the *C*_44_ constant is negative, indicating mechanical instability in the cubic γ-SiB_6_ structure. This is in agreement with previous calculations, where γ-phase is found to be mechanically unstable [9].

In the case of the α-SiB_6_ phase, these are the first reports of the elastic constants, calculated using LDA (Table 4) and GGA (Table A2). There are four conditions for hexagonal phases [68].

*C*_11_ > |*C*_12_|; 2*C*_13_^2^ < *C*_33_(*C*_11_ + *C*_12_); *C*_44_ > 0; *C*_66_ > 0,

and *C*_66_ constant has been calculated with *C*_66_ = 1/2(*C*_11_ − *C*_12_) [49,50]. The *α*-SiB_6_ modification, calculated in a hexagonal setting, appears mechanically stable, and was calculated using the LDA method. Moreover, we show additional *C*_31_ and *C*_15_ elastic constants for the rhombohedral unit cell.

Finally, we move to the *β*-SiB_6_ phase with a lower orthorhombic symmetry and a larger number of independent elastic constants. They were all calculated using both the LDA and GGA approach, and concur with previous theoretical data [17] (Table 4 and Table A2). There are six conditions for orthorhombic crystal system [68]:

*C*_11_ > 0; *C*_11_*C*_22_ > *C*_12_^2^;

*C*_11_*C*_22_*C*_33_ + 2*C*_12_*C*_13_*C*_23_ − *C*_11_*C*_23_^2^ − *C*_22_*C*_13_^2^ − *C*_33_*C*_12_^2^ > 0;

*C*_44_ > 0; *C*_55_ > 0; *C*_66_ > 0;

The calculated *β*-SiB_6_ modification appears mechanically stable regardless of the DFT method applied, which is in agreement with previous calculations, where it has been found as mechanically and dynamically stable [17].

In this study, we have calculated bulk modulus *B*, shear modulus *K*, Young’s modulus *E*, Poisson’s ratio *v*, and Pugh’s criterion *B/K,* for three SiB_6_ modifications using LDA and GGA approximations. The value of the bulk modulus has been predicted between 146.44–153.43 GPa using GGA and 154.59–169.21 GPa using LDA for all three SiB_6_ phases (Table 5). This is in good agreement with other theoretical studies on the Si–B compound, where the bulk modulus was calculated in the range between 118 and 183 GPa [9,11,17]. Shear modulus has been predicted in a wide range from 22.21 to 93.88 GPa using GGA, and 23.63–92.09 GPa using the LDA method. This is in reasonable agreement, since literature data on other silicon borides show an even wider span of calculated B values (39.6–157.4 GPa [9,11,70]). A similarly calculated range for the *E* modulus using both GGA and LDA is comparable with the 150–358.8 GPa computed for other Si–B compounds (Table 5) [9,11,70].

Apart from the elastic moduli (*B, K,* and *E*) shown above, Poisson’s ratio is another important mechanical property. Poisson’s ratio, ν, is the negative ratio of the lateral or transverse strain to the axial strain in tensile loading and is thus interrelated with Young’s modulus E [71,72]. Our DFT calculations show ν in the range between 0.24 and 0.43, regardless of the computational approach (Table 5), and are in a reasonable agreement with the values of 0.17–0.35 reported for various silicon borides [9,11,70]. Moreover, Poisson’s ratio provides information about the ductility/brittleness of the materials. If the *v* value is smaller than 0.26, the material will have brittle behavior; otherwise, it is ductile. It appears that *β*-SiB_6_ has a brittle character, while α- and γ-phase appear to be ductile, after using both GGA and LDA methods (Table 5).

This can be further investigated with Pugh’s forecast material delay/brittle empirical criterion (B/K) (Table 5) [73]. The critical value of Pugh’s criterion, which separates ductile and brittle materials, is around or higher than 1.75. If higher than this value, the material behaves in a ductile manner, otherwise, the material behaves in a brittle manner [74]. Again, according to the B/K relationship, the *β*-SiB_6_ phase has a brittle character, while α- and γ-modifications show ductile behavior regardless of the computational approach (Table 5).

**Figure 5:**
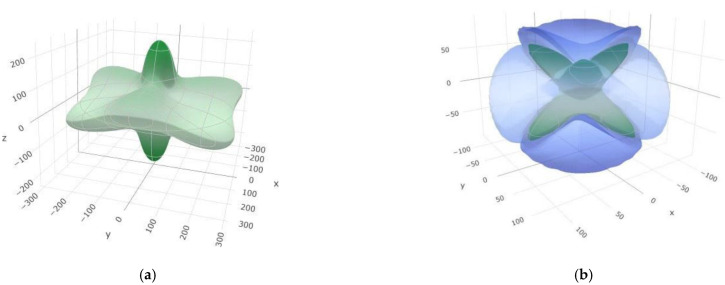
3D contour plots of anisotropic surface figures of (**a**) Young’s modulus and (**b**) shear modulus for the α-SiB_6_-type of structure.

**Figure 6:**
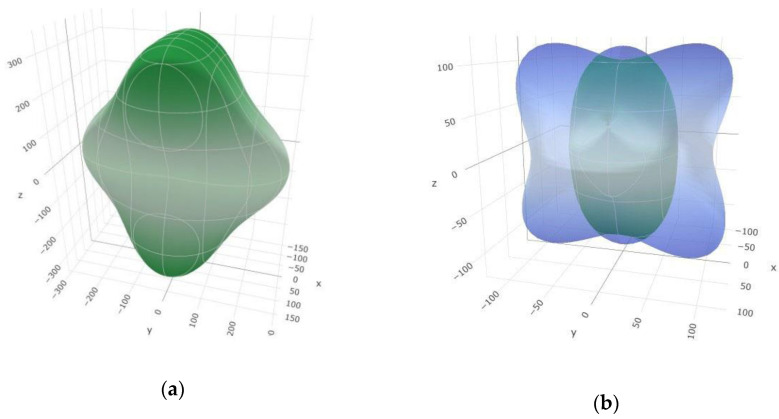
Visualization of the 3D contour plots of anisotropic surface figures: (**a**) Young’s modulus; (**b**) shear modulus for the β-SiB_6_ modification.

**Figure A1:**
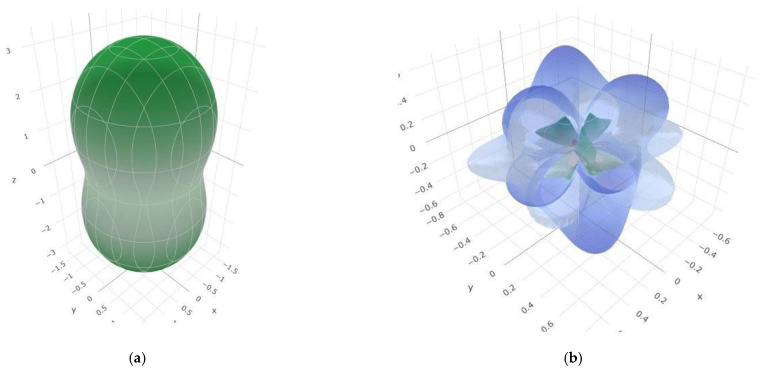
3D contour plots of anisotropic surface figures of (**a**) linear compressibility and (**b**) Poisson’s ratio for the α-SiB_6_-type of structure.

**Figure A2:**
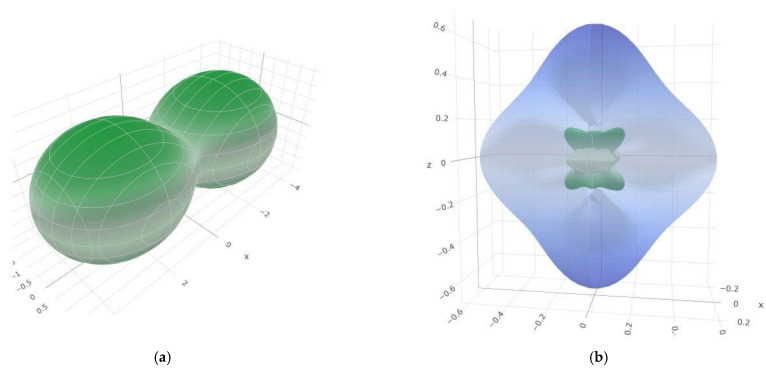
3D contour plots of anisotropic surface figures of (**a**) linear compressibility and (**b**) Poisson’s ratio for the β-SiB_6_-type of structure.

In order to illustrate Young’s and shear modulus anisotropy, we have plotted the 3D anisotropic surface figures of the *K* and *E* modulus under the spherical coordinates for the α-phase (Figure 5). The content of anisotropy depends on the deviation degree from the spherical shape. The degree of deviation between the sphere and the surfaces which we obtained suggests a high degree of elastic anisotropy, especially in the shear modulus (Figure 5b), while Young’s modulus shows the smallest deviation from the sphere in the *xy* plane (Figure 5a). Moreover, 3D contour plots of the anisotropic surface figures of Young’s and shear modulus for the β-SiB_6_ modification have been performed (Figure 6). In contrast to the α-phase, the β-SiB_6_ shows a lower degree of elastic anisotropy in Young’s modulus, since it is more spherical in all three planes (Figure 6a), while shear modulus is mostly spherical in the *xy* plane (Figure 6b). In addition, visualization and anisotropic analysis of the linear compressibility and Poisson’s ratio in 3D for both α- and β-phases have been presented in Appendix A. A smaller deviation degree from the spherical shape has been found for the linear compressibility, while Poisson’s ratio largely deviates, indicating a high degree of elastic anisotropy (Figure A1 and Figure A2).

## 4. Conclusions

Structure prediction and mechanical properties investigations of silicon hexaboride on an ab initio level have been performed. Data-mining-based searches over the ICSD database combined with the PCAE method produced 44 structure candidates, which, after full structural optimization using two DFT methods (LDA and GGA), have been reduced to four final SiB_6_ structures, regardless of the computational approach. Two novel structures are denoted as the α-SiB_6_-type, appearing in the hexagonal space group *R-3mH* (no. 166), and as δ-SiB_6_-type that crystallizes in the space group *P3m1* (no. 156) have been predicted, for which there are no previous experimental or theoretical data. Our DFT calculations on the experimentally known cubic γ-SiB_6_ and recently proposed orthorhombic β-SiB_6_ phase are in very good agreement with previous findings. Elastic and mechanical properties of the predicted structures were investigated in the next phase. There is a limited number of such investigations and, in this study, the elastic constants for the most relevant silicon hexaboride modifications have been calculated using the GGA-PBE and LDA-PZ approaches, and were compared to previous theoretical data where found.

Calculated elastic constants are in good agreement with available theoretical data and show α-SiB_6_ and β-SiB_6_ as mechanically stable. Besides, we have calculated bulk modulus *B*, shear modulus *K*, Young’s modulus *E*, Poisson’s ratio *v*, and Pugh’s criterion *B/K* for various SiB_6_ modifications using LDA and GGA approximations, and our results concur with other theoretical studies on the related Si–B compound. From the calculated Poisson’s ratio and Pugh’s criterion (B/K), it appears that *β*-SiB_6_ will have brittle character, while α- and γ-phase appear to be ductile using both GGA and LDA methods. In addition, we have plotted the 3D anisotropic surface figures of *K* and *E* modulus under the spherical coordinates for the α- and β-phase. We believe that our results could potentially have a great impact on the industrial and technological applications of silicon boride-based materials.

## Figures and Tables

**Table 1:** The total energy values (in E_h_) and relative energies (compared to the global minimum α-SiB_6_ structure taken as the zero of energy in E_h_) of the SiB_6_ modifications obtained from data-mining-based searches and local optimization on the GGA-PBE and LDA-PZ level of calculations.

Modifications	Total Energy		Relative Energy	
	GGA-PBE (E_h_)	LDA-PZ (E_h_)	GGA-PBE (E_h_)	LDA-PZ (E_h_)
α-SiB_6_	−438.2996	−435.9498	0	0
β-SiB_6_	−438.2990	−435.9432	−0.0006	−0.0066
γ-SiB_6_	−438.2009	−435.8422	−0.0987	−0.1076
δ-SiB_6_	−438.1313	−435.7806	−0.1683	−0.1692

**Table 2:** Full structural details (modifications, space group, unit cell parameters, and atomic positions) for predicted SiB_6_ modifications obtained from data-mining-based searches and local optimization on the GGA-PBE level.

Modification and Structure Type	Space Group	Cell Parameters	Position of Atoms
α-SiB_6_ PB_6_-type	*R-3mH* (no. 166)	a = 6.164 c = 12.079	Si 0.000000 0.000000 0.898652
			B 0.150405 0.300810 0.527650
			B –0.104630 −0.209260 0.618452
β-SiB_6_ *Cmca*-B_6_Si	*Cmce* (no. 64)	a = 5.894 b = 11.184 c = 8.420	Si 0.000000 0.825124 0.876686
			B 0.734869 0.925007 0.973036
			B 0.341293 0.039457 0.830441
			B 0.000000 0.650575 0.938946
			B 0.000000 0.396919 0.849121
γ-SiB_6_ CaB_6_ type, SiB_6_-*cubic*	*Pm-3m* (no. 221)	a = 4.161	Si 0.000000 0.000000 0.000000
			B 0.800175 0.500000 0.500000
δ-SiB_6_	*P3m1* (no. 156)	a = 3.503 c = 6.407	Si 0.000000 0.000000 0.000000
			B 0.831905 0.663809 0.465145
			B 0.999478 0.499739 0.726170

**Table 3:** Calculated unit cell parameters of the SiB_6_ modifications compared with the previous experimental and theoretical results where available. Local optimizations were performed within the DFT (GGA) and (LDA) approximations.

Modification	Experiment/ Theory (Å)	GGA-PBE (Å)	LDA-PZ (Å)
α-SiB_6_	*n.a.*	a = 6.164 c = 12.079	a = 6.160 c = 11.690
β-SiB_6_	a = 5.8443 b = 11.0988 c = 8.3697 ^a^	a = 5.894 b = 11.184 c = 8.420	a = 5.790 b = 11.053 c = 8.322
γ-SiB_6_	a = 4.130 ^b^ a = 4.13 ^c^	a = 4.161	a = 4.109
δ-SiB_6_	*n.a.*	a = 3.503 c = 6.407	a = 3.465 c = 6.059

^a^ Theo. PW/PPs-GGA-PBE [17], ^b^ Exp. [8], ^c^ Theo. PW/PPs-GGA-PBE [9].

**Table 4:** Calculated elastic constants *Cij* (GPa) for various SiB_6_ modifications using LDA approximation and compared to previous calculations.

*Cij* (GPa)	LDA		
	*α-SiB*_6_	*β-SiB*_6_	*γ-SiB*_6_
*C*_11_	380.48	165.19 205 ^a^	404.76 402.6 ^b^
*C*_12_	144.82	97.46 79 ^a^	32.55 19.31 ^b^
*C*_13_	69.75	109.01 97 ^a^	-
*C*_15_	28.99	-	-
*C*_21_	-	101.32	-
*C*_22_	-	352.39 338 ^a^	-
*C*_23_	-	66.19 57 ^a^	-
*C*_31_	69.94	112.28	-
*C*_32_	-	68.81	-
*C*_33_	249.68	409.08 397 ^a^	-
*C*_44_	39.80	102.57 100 ^a^	−11.62 −4.13 ^b^
*C*_46_	-	-	-
*C*_55_	-	117.45 123 ^a^	-
*C*_66_	117.83	63.65 72 ^a^	-

^a^ [17], ^b^ [9].

**Table 5:** Calculated bulk modulus B (GPa), shear modulus K (GPa), Young’s modulus E (GPa), Poisson’s ratio *v*, and Pugh’s criterion *B/K,* for various SiB_6_ modifications using LDA and GGA approximations.

Mechanical Property	LDA			GGA		
	*α-SiB*_6_	*β-SiB*_6_	*γ-SiB*_6_	*α-SiB*_6_	*β-SiB*_6_	*γ-SiB*_6_
*B*	169.21	154.59	156.63	153.43	147.22	146.44
*K*	71.55	92.09	23.63	47.22	93.88	22.21
*E*	188.12	230.49	67.49	128.48	232.26	63.42
*v*	0.32	0.25	0.43	0.36	0.24	0.43
*B/K*	2.36	1.68	6.63	3.25	1.57	6.59

## Data Availability

Additional data may be requested from the first author.

## Appendix A

**Table A1:** Full structural details (modifications, space group, unit cell parameters, and atomic positions) for SiB_6_ modifications obtained from data-mining-based searches and local optimization on the LDA-PZ level.

Modification and Structure Type	Space Group	Cell Parameters	Position of Atoms
α-SiB_6_ PB_6_-type	*R-3mH* (no. 166)	a = 6.160 c = 11.690	Si 0.000000 0.000000 0.868964 B 0.149903 0.299806 0.528563 B −0.103858 −0.207715 0.618537
β-SiB_6_ *Cmca*-B_6_Si	*Cmce* (no. 64)	a = 5.790 b = 11.053 c = 8.322	Si 0.000000 0.824075 0.875433 B 0.736220 0.925098 0.973414 B 0.341461 0.038884 0.830830 B 0.000000 0.650086 0.938827 B 0.000000 0.396399 0.850014
γ-SiB_6_ CaB_6_ type	*Pm-3m* (no. 221)	a = 4.109	Si 0.000000 0.000000 0.000000 B 0.799534 0.500000 0.500000
δ-SiB_6_	*P3m1* (no. 156)	a = 3.465 c = 6.059	Si 0.000000 0.000000 0.000000 B 0.831926 0.663851 0.439282 B 0.999194 0.499597 0.712537

**Table A2:** Calculated elastic constants *C**ij* (GPa) for various SiB_6_ modifications using LDA approximation and compared to previous calculations.

*Cij*	GGA-PBE (GPa)		
	α-SiB_6_	β-SiB_6_	γ-SiB_6_
*C*_11_	80.39	187.47	362.06
*C*_12_	−90.35	83.66	38.62
*C*_13_	207.40	100.87	38.62
*C*_15_	10.28	-	-
*C*_21_	-	86.67	-
*C*_22_	-	322.35	-
*C*_23_	-	53.39	-
*C*_31_	323.26	97.85	-
*C*_32_	-	54.68	-
*C*_33_	132.77	379.45	-
*C*_44_	39.99	96.55	−8.70
*C*_46_	-	-	-
*C*_55_	-	118.88	-
C_66_	85.37	64.39	-

## Appendix B

An all-electron basis set based on Gaussian-type orbitals was employed and is given below. In the case of Silicon a *[5s4p1d]* basis set was used [39,40], and *[3s2p1d]* basis set was used in the case of boron [41,42,43].

5 4 #B_BN_ Doll_2008

0 0 6 2. 1.

2.082E + 03 1.850E-03

3.123E + 02 1.413E-02

7.089E + 01 6.927E-02

1.985E + 01 2.324E-01

6.292E + 00 4.702E-01

2.129E + 00 3.603E-01

0 1 2 3.0 1.0

2.282E + 00 −3.687E-01 2.312E-01

4.652E-01 1.199E + 00 8.668E-01

0 1 1 0.0 1.0

0.16 1.000E + 00 1.000E + 00

0 3 1 0.0 1.0

0.5 1.0

14 6 #Si_86-311G**_pascale_2005

0 0 8 2. 1.0

87645.8 0.000237

12851.8 0.00192

2786.28 0.0109

728.043 0.0496

219.516 0.1668

75.9006 0.363

29.4602 0.4051

11.9891 0.1504

0 1 6 8. 1.0

165.958 −0.00884 0.00909

39.3727 −0.0859 0.0601

12.7112 −0.0712 0.1952

4.7177 0.4147 0.3384

1.8482 0.6168 0.3006

0.7 0.1154 0.0648

0 1 3 4. 1.

4.1752 −0.0199 −0.0087

1.4472 −0.1864 −0.00438

0.48 0.0967 0.2207

0 1 1 0. 1.

0.25 1. 1.

0 1 1 0. 1.

0.13 1. 1.

0 3 1 0. 1.

0.13 1.
